# Ultraconservative and Aesthetic Approach for Rehabilitation of Complicated Crown Fracture of Tooth

**DOI:** 10.7759/cureus.26259

**Published:** 2022-06-23

**Authors:** Meghna Dugar, Anuja Ikhar, Pradnya Nikhade, Manoj Chandak, Saurabh Rathi

**Affiliations:** 1 Conservative Dentistry and Endodontics, Sharad Pawar Dental College And Hospital, Datta Meghe Institute of Medical Sciences, Wardha, IND; 2 Dentistry, Sharad Pawar Dental College And Hospital, Datta Meghe Institute of Medical Sciences, Wardha, IND; 3 Conservative Dentistry and Endodontics, VYWS (Vidarbha Youth Welfare Society) Dental College & Hospital, Amravati, IND

**Keywords:** crown fracture, conservative approach, trauma, reattachment, fracture

## Abstract

Dental trauma is well considered to be a general health issue. The prevalence of tooth fracture in the anterior region looks for a conservative approach to treatment. Currently, a novel and ultraconservative approach is the most popular treatment option for the rehabilitation of the fractured tooth. Fragment removal and proper repositioning followed by retention of the tooth’s fragments using adhesive techniques provides added benefits such as aesthetics, restoration of surface gloss, function, shape, and texture, as well as maintaining the tooth’s position and original morphology. Satisfactory results with less tooth destruction and utmost original anatomy preservation were seen during follow-ups. This approach is seen to be ultraconservative, safe, and pleasing aesthetically. However, careful planning, preoperative assessment, and case selection are the prerequisites in order to attain a favourable prognosis for the long term.

## Introduction

Traumatic dental injuries can be referred to as physical injuries present in the dento-alveolar region. They may be of a mild, moderate or severe type, varying on the kind of trauma as well as the involved tissues [1]. Some traumatic injuries are categorized as an emergency in endodontics and, hence, require urgent attention [2]. These injuries are most common in preschool children (25%) and adolescents (33%) as a result of traffic and sports accidents [3]. If the line of fracture is in the subgingival region then surgical as well as orthodontic approaches should be considered [4]. Even though the respective tooth could be extruded orthodontically, reattachment of the fractured segment could be faster by surgical flap elevation [5]. In the presence of the tooth fragment, the primary goal should be restoration with the use of tooth fragments only. Proper repositioning followed by retention of tooth fragment using adhesive technique provides added benefits such as restoration of aesthetics, surface gloss, function, shape, and texture, as well as maintaining the tooth’s position and original morphology. As it is economically effective and only a short time is required, it can positively enhance the psychology of the patient, thereby increasing the treatment acceptability [6].

The reattachment concept started in the year 1964 when Eidelman and Chosak utilized a cast post as well as a regular cement to attach the segment of the crown [7]. Recently, with advancements in materials used for restoration, placement techniques as well as adhesive protocols, reattachment is performed. Tennery first used the acid etching technique to reattach the fractured tooth [8]. Eliminating bacteria from the canal remains a primary objective in endodontic therapy; therefore, a combination of treatment techniques has been used for complete bacterial elimination. Recently, dental lasers have shown to provide a significant advantage in canal disinfection over some conventional methods.

Complicated fractures of the crown involving enamel, dentin, as well as pulp have a major contribution to overall dental injuries. Immediate clinical consideration is required in cases of fractured anterior teeth, which if left untreated could cause damage to the dentition along with a psychomatic impact on the patient [9]. Root canal therapy with subsequently fractured segment reattachment and reinforcement of fibre post is a viable option for treating the case of complicated crown fracture. According to literature, when resin cement is used for luting the fibre post, it leads to an increase in retention of the segment and provides a “monoblock effect”. The current report presents a complicated crown fracture case, which was managed successfully by reattachment of the tooth and the fractured segment.

## Case presentation

**Figure 1:**
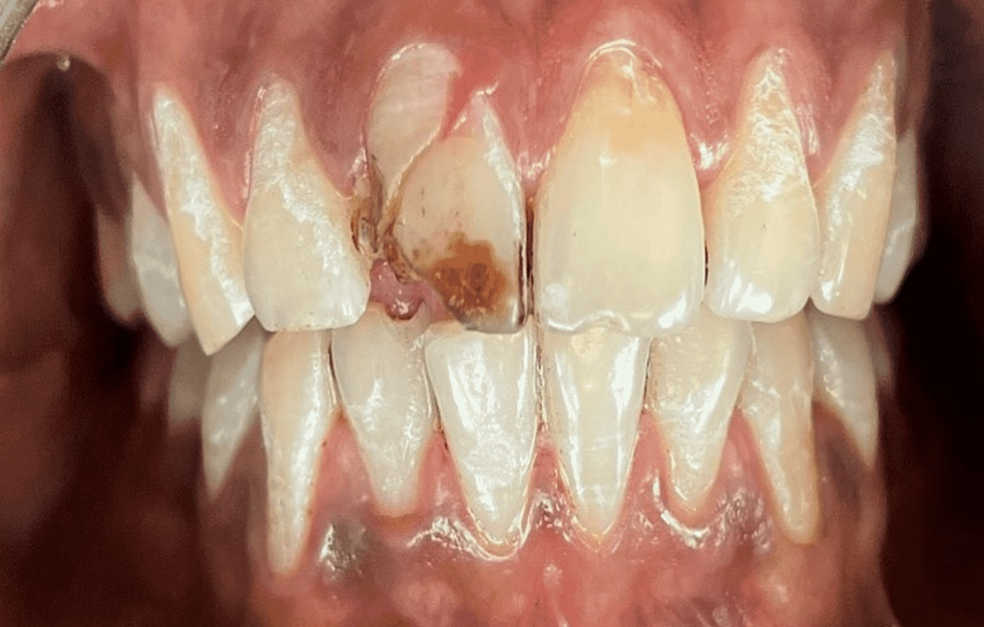
Intraoral Image with respect to 11

A 20-year-old boy arrived for treatment with a complaint of maxillary right fractured central incisor due to a fall from a vehicle. A part of the fractured tooth was lost during the fall, leaving behind the other part of the fractured tooth mobile, yet attached palatally. No discolouration was seen in the coronal structure. On clinically examining the tooth structure, the fractured fragment was seen to be mobile (Figure 1).

**Figure 2:**
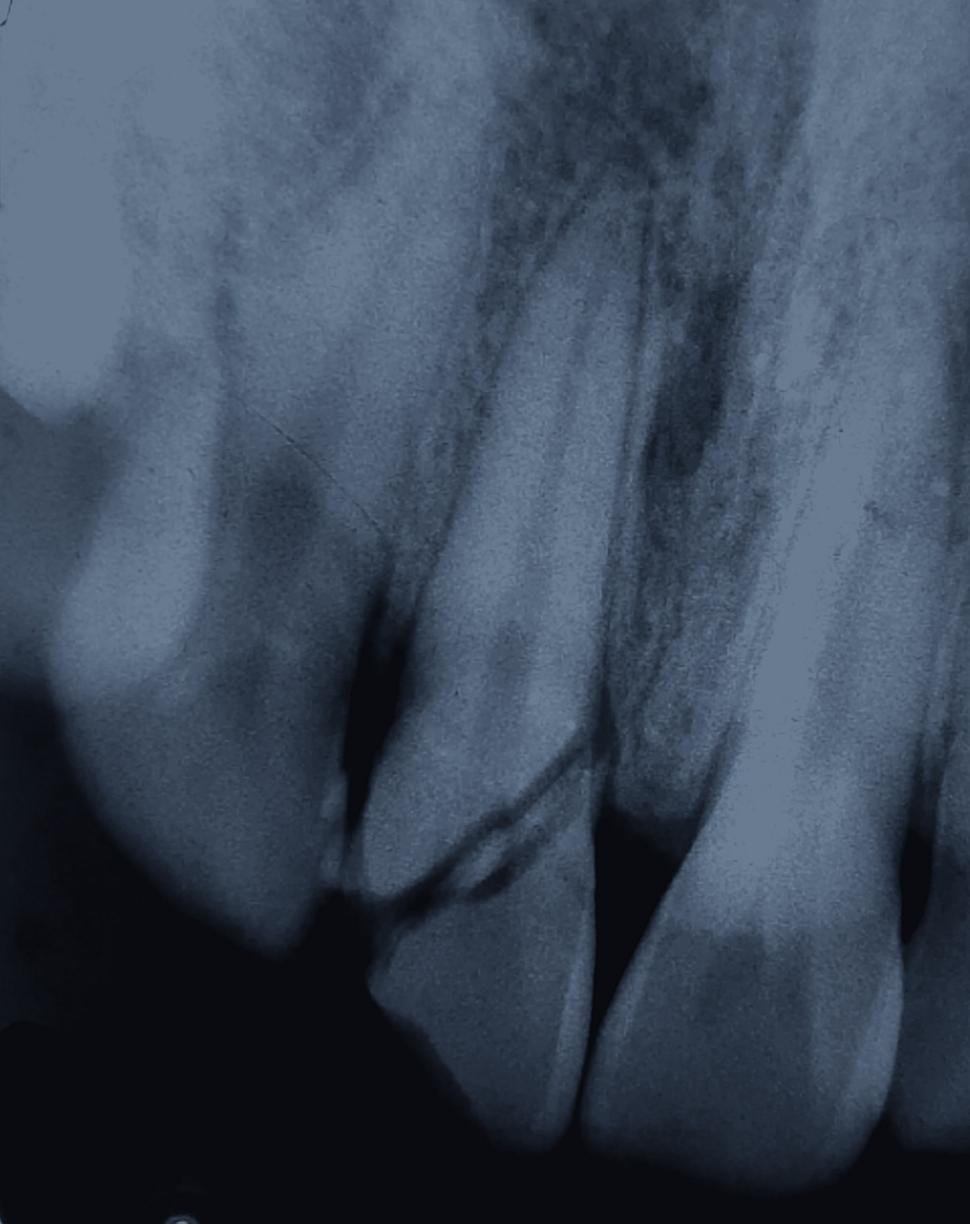
Intraoral periapical radiograph with respect to 11

The respective tooth was associated with mild and dull aching pain. A radiograph was taken, which revealed a complicated crown fracture, where the fracture line was appreciated running from mesial to distal side obliquely from cemento-enamel junction towards the middle third. It also revealed the mesial drift of the coronal part of the tooth, only having attachment palatally. The radiograph also revealed exposure of pulp, which confirmed the need for endodontic treatment. Periapical radiographs depicted the periodontal ligament space to be intact, with no root fracture in the tooth’s relation as well as complete formation of root (Figure 2).

**Figure 3:**
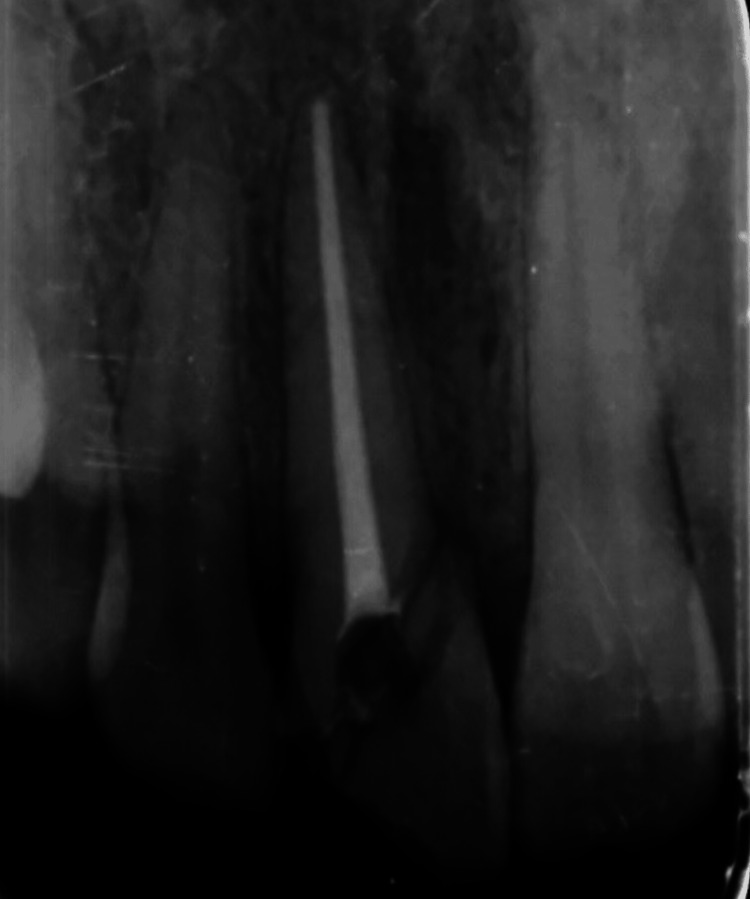
Obturation done in 11

The parents of the patient were familiar with the risk associated with the loss of a tooth. After having detailed knowledge about the procedure, keeping in mind the age factor of the patient, they decided to opt for re-attachment of the fragment by accepting the limitations and risk associated with an attempt to save the tooth. Local anesthesia (i.e, 2% lidocaine of 1.0 cc with a ratio 1: 80,000 epinephrine) was administered and the mobile segment was stabilized temporarily with luting type of glass ionomer cement. Root canal therapy was performed using the standard step-back preparation technique and cold lateral condensation obturation technique in relation to 11 (Figure 3).

**Figure 4:**
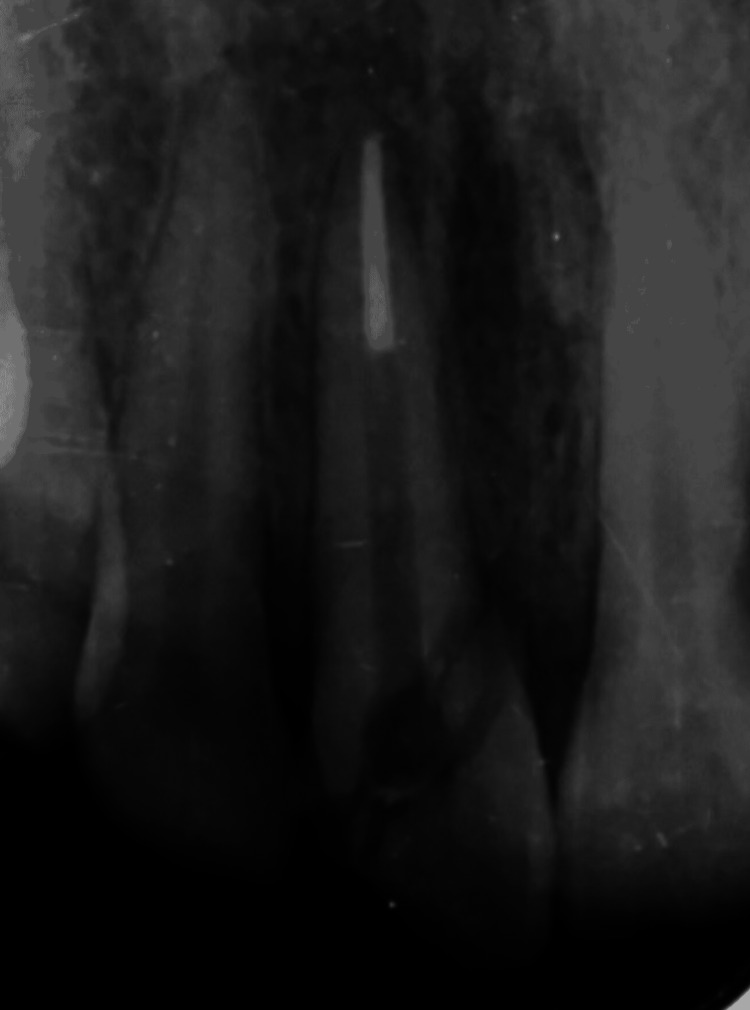
Post space preparation done with 11

After this, the fractured fragment was removed carefully and chlorhexidine 2% solution was used for cleaning; thereafter, isotonic saline solution was used to store it. Vents were made in the crown through and through and groove was also made in the dentin internally. Post-space preparation was done using Peeso reamers and Gates Glidden (GG) drills (Figure 4).

**Figure 5:**
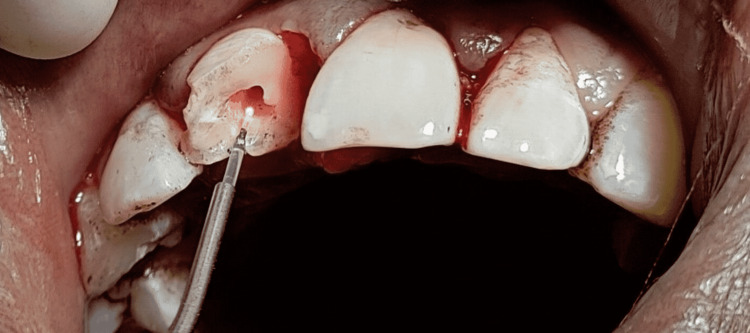
Dental laser used to disinfect the canal with 11 before post placement

Elevation of full-thickness buccal and palatal mucoperiosteal flaps with a 15 BP blade was done in order to precisely evaluate its respective relation to the crestal bone as well as to enhance the accessibility of the fracture line to the extent of the gingiva. Fibre post of 1.1 mm diameter (Reforpost glass fiber, Angelus Dental, Londrina, Brazil) of aesthetic type was chosen. For canal drying, paper points were used. Biolase dental laser (Biolase, Irvine, California, United States) was used to disinfect the canal before placement of the post. Four cycles of the laser beam were used; five seconds in each cycle (Figure 5). 

**Figure 6:**
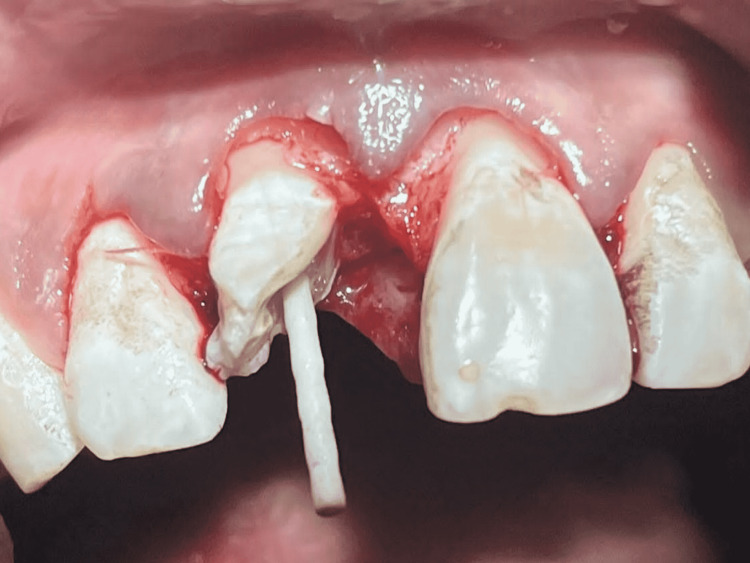
Post placement done with 11

Post space prepared was etched using 37% of phosphoric acid. Thorough rinsing was done and excess water removal was done using a pellet of cotton. Then adhesive application on the post as well as on the etched surface was done after air-thinning and light-curing it for 10 seconds. Resin cement was used to lute the post with a coronal portion of 2-2.5 mm extending to the pulpal chamber (Figure 6).

**Figure 7:**
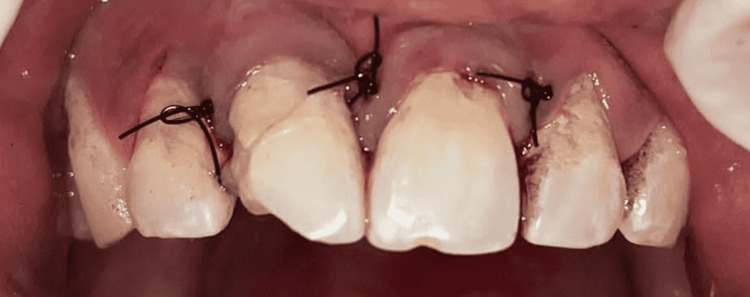
Sutures placed

After achieving hemostasis, luting of the post was performed with dual-cure resin and the tooth segment was reattached with the help of resin cement. Repositioning and suturing of the flap were done after this (Figure 7).

**Figure 8:**
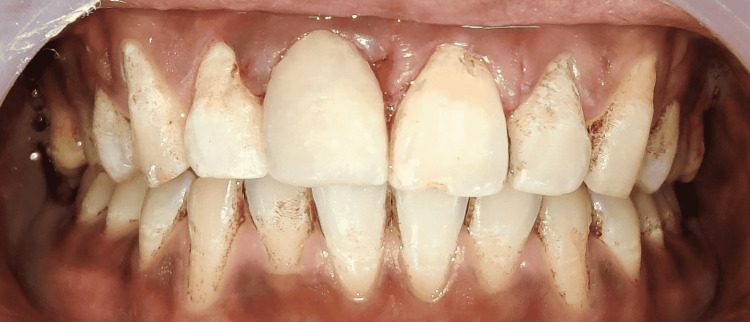
Composite buildup with 11

Proper rehabilitation was done by performing composite buildup and restoring the original anatomy of the tooth (Figure 8).

**Figure 9:**
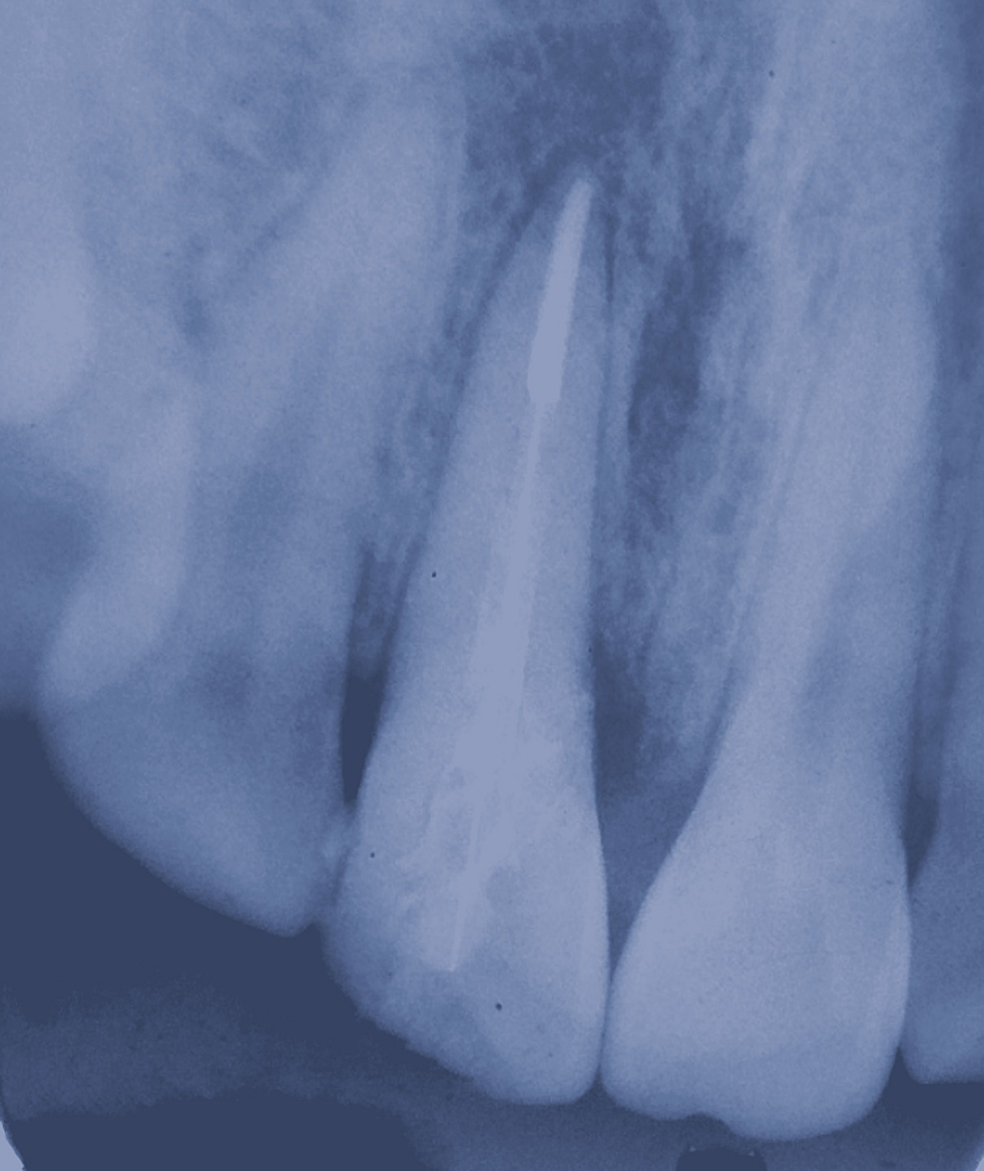
Intraoral periapical radiograph at three-month follow-up

Periodic review was done for the patient and it was noted that both restorative, as well as endodontic treatments, remained acceptable clinically at the three-month follow-up (Figure 9).

**Figure 10:**
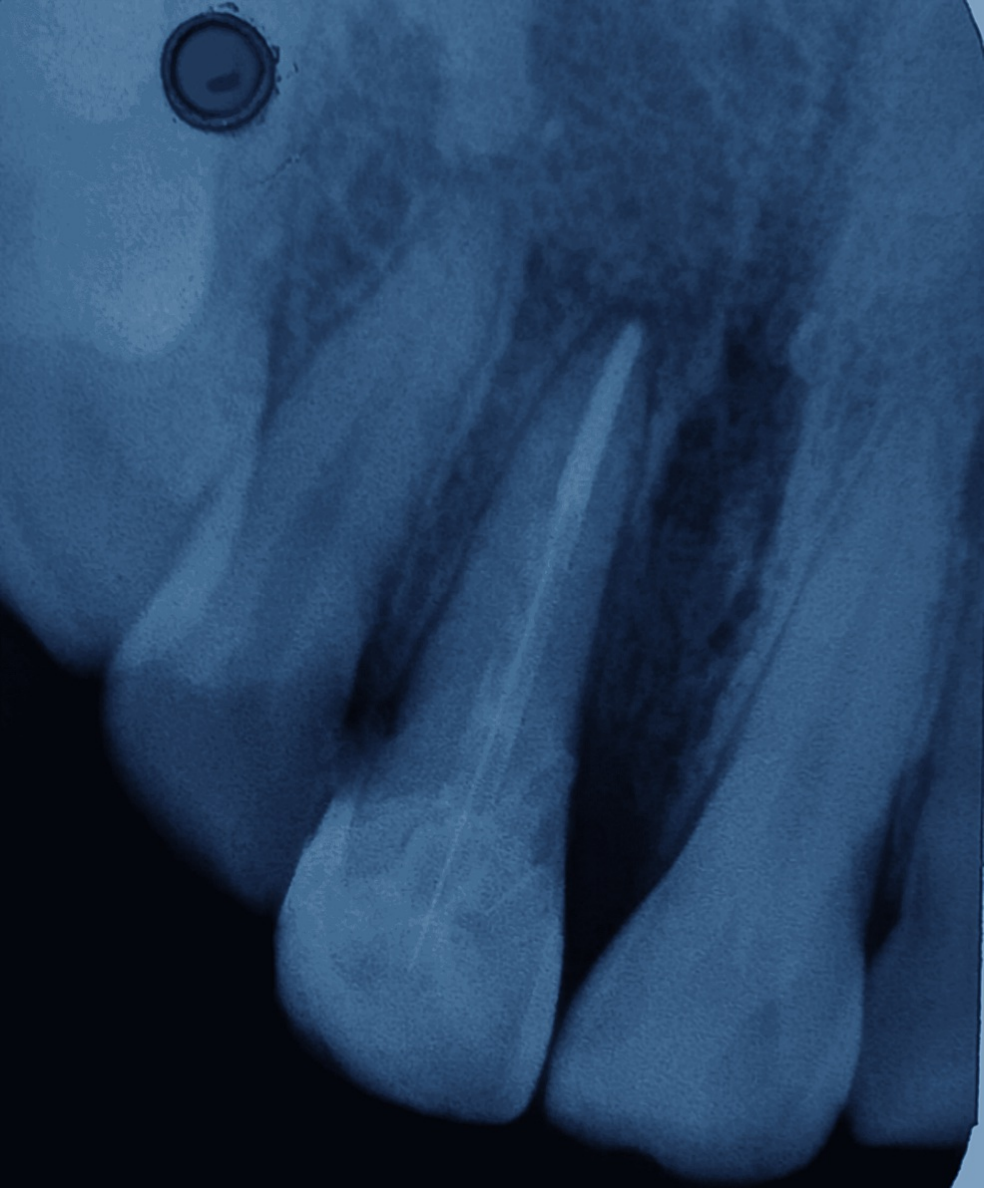
Intraoral periapical radiograph at six-month follow-up

Both clinical, as well as radiographic images, were also done at six months and they showed favourable healing (Figure 10).

## Discussion

Fracture of teeth in the anterior region is not a very uncommon consequence of trauma and is of utmost importance to the patient because it is aesthetically involved. Management in such cases should be very conservative with a positive resulting outcome [10]. In this case, the management of incomplete crown fracture is well described with a simple and a conservative approach.

Partial coverage crowns, full coverage crowns, composite resins, and laminate veneers are some of the initial methods that were helpful in restoring the teeth with fracture. However, these have various disadvantages such as being time-consuming, not very conservative, and expensive. In the year 1964, Eidelman and Chosack first elaborated on restoration of fractured teeth with the tooth fragment and suggested that this could be the best possible way to establish the natural shape, contour, surface texture, fragment’s colour, as well as occlusal alignment. Multiple case reports in literature have well portrayed that fractured tooth fragment could be reattached for managing coronal fracture in anterior teeth with the availability of fractured segment. It is obligatory to keep the fragment hydrated for maintaining natural esthetic presentation of the tooth along with enough bond strength. Therefore, after the fractured fragment separation, sterile isotonic saline was used for hydration restoration [11].

The case discussed here features the line of fracture in the mesio-palatal direction and subgingivally but not at the expense of the biological width. On further clinical examination, since there was only minimal biological width invasion, osteotomy procedure was not necessary. For perfect fragment and tooth approximation, mucoperiosteal flap (full thickness) was raised on both palatal aspects as well as buccal side. Amongst all the techniques including different material, the most appropriate option is fibre post of tooth colour along with resin cement. With fibre post there is good aesthetics, appropriate elastic modulus, lesser chairside time, better post and cement bonding, and minimum tissue removal. The literature also suggests the importance of the use of fibre post in cases of fractured teeth owing to the fact that it helps in interconnecting the fractured fragment and tooth and minimizes the stress load on the reattached fragment and many other advantages such as a suitable elastic modulus, aesthetics, good bonding between post and cement, lower chairside time, and minimal tissue removal [12].

After the post space preparation, canal disinfection was done using a dental laser. Gordon et al. studied the ability of this laser to disinfect dentin and concluded that bacterial recovery decreased with increase in laser duration or power. Application of two minutes of laser resulted in better disinfection than with sodium hypochlorite (NaOCl) usage alone.

In addition to the post space preparation, a leeway vent in coronal part of separated segment was created for better flowability of excessive cement without creating hydrostatic pressure. Also, an external chamfer in the labial enamel was made for strengthening the restoration and prevent microleakage. According to Reis et al., reattachment of coronal fragments with that of remaining part of the tooth, along with the use of an internal groove/overcontour technique provides increased fracture strength to restored teeth by 97.2% and 90.5%, respectively [13]. In 2012, Srilatha et al. in her study concluded that better strength recovery was seen with overcontouring technique, attributing to more adhesion area hence better distribution of force [14]. Hence, in this technique there is reinforcement of the segments restored leading to a high durability of the restoration. Pusman et al. showed in a study that along with bonding, flowable composite performed better in shear bond strength because of its adequate flow through the attachment site [15]. The fragment was therefore approximated with flowable composite resin layer and with microhybrid composite thereafter. After this, microfilled composite was veneered on labial surface of the teeth for the purpose of inhibiting microleakage because of enamel cracks. Surface finishing and contours of subgingival restoration decides the prognosis of respective tooth. With plaque and diet control, the presentation of the restoration is maintained. As far as retention is concerned, reattachment also compulsorily rely on bonding of restorations. Along with a demand of adhesives in dentistry, many types of adhesives as well as procedures have been practiced for producing favourable bond at restoration and tooth interface. In this, the etch and rinse types, with approximately 30 MPa bond strength, are the gold standard. This report, hence, describes a method for complicated/partial fracture treatment. Although convincing, it is however not the universal approach for fracture treatment.

## Conclusions

The case presented showed an alternative approach to treatment for reattaching a fractured tooth fragment without its removal. This approach has been seen to be ultraconservative, safe, and pleasing aesthetically. However, careful planning, preoperative assessment, and case selection are the prerequisites in order to attain a favourable prognosis for the long term.
